# How do college coaches in the United States identify youth female and male soccer players?

**DOI:** 10.1371/journal.pone.0331134

**Published:** 2025-09-12

**Authors:** Matthew Andrew, Sam Barraclough, Andrew O. Triggs, James H. Dugdale, Adam Kelly, Matthew J. Reeves

**Affiliations:** 1 Department of Sport and Exercise Sciences, Manchester Metropolitan University Institute of Sport, Manchester, United Kingdom; 2 UCFB Manchester Campus, Manchester, United Kingdom; 3 Research Institute for Sport and Exercise Sciences, Liverpool John Moores University, Liverpool, United Kingdom; 4 School of Applied Sciences, Edinburgh Napier University, Edinburgh, United Kingdom; 5 Faculty of Health, Education and Life Sciences, Birmingham City University, Birmingham, United Kingdom; 6 Research Centre for Applied Sport, Physical Activity & Performance, University of Central Lancashire, Preston, United Kingdom; 7 Department of Physical Sport Sciences, College of Sports Sciences & Physical Activity, Princess Nourah bint Abdulrahman University, Riyadh, Saudi Arabia; Universidade Federal de Goias, BRAZIL

## Abstract

Talent identification (TI) in soccer is a complex and multifactorial process within the context of collegiate sport in the United States, where coaches must assess performance-ready athletes often under strict regulatory and resource constraints. Despite the critical role college coaches play in bridging youth to professional soccer, little is known about their evaluative priorities during recruitment. This study examined how soccer coaches from female and male NCAA Division I and II programs perceived the importance of various player attributes and scouting methods in the TI process. A total of 178 college soccer coaches completed a survey assessing perceptions across seven attribute categories (technical, physical, psychological, game intelligence, social, other, and coach-specific) and common scouting methods. Bootstrapped trimmed means, effect sizes, and inter-rater agreement (r_wg_) were used to analyse the coaches’ ratings of importance across attributes and to assess for differences in coach perceptions within the female and male programs. Results showed coaches across female and male programs rated technical proficiency, coachability, decision-making, and work rate as critically important. College-specific soccer knowledge was the most highly valued coach attribute, while live match observation was the most preferred scouting method. Sex-based differences were generally minimal, although emerged in perceptions of physical and social attributes, with coaches of female players placing greater emphasis on communication and agility. Results highlighted a shared prioritisation of technical and psychological qualities in college TI, with contextual differences influenced by sex and program structure. These insights support the development of more aligned and evidence-informed TI strategies in collegiate soccer environments.

## Introduction

Talent identification (TI) in soccer represents a challenging yet critical process, identifying players who not only demonstrate current performance competences but possess long-term potential for success at professional and international levels [1,2]. Due to the non-linear trajectory of talent development (TD) and the multidimensional skills required to excel in soccer, evaluating potential is a complex undertaking [3,4], with no agreed “gold standard” method of or for TI. Traditionally, TI has relied heavily on coaches and scouts observing players during practice and competitive play, with subsequent judgements often shaped by subjective impressions rooted in experience and intuition [5,6]. While such methods remain foundational, this reliance on the ‘coach’s eye’ and the accompanying risk of biased or inaccurate assessments, has prompted calls for more of an evidence-informed approach [7,8].

To mitigate the limitations of solely subjective evaluations and interpretations, and the fundamental risk of coach perceptions incorrectly predicting future performance, a growing body of research has offered objective insights into the various attributes linked to future success in soccer. Over the past three decades, these efforts have focused on the predictive validity of physical, technical, game intelligence, and psychological skills, as well as sociological factors [1,2]. Findings have consistently indicated that advanced technical abilities, including ball control, passing, dribbling, and shooting, are indicators of progression to professional levels. Indeed, longitudinal studies from the Netherlands [9], Finland [10], and Germany [11,12] have demonstrated that players exhibiting higher proficiency in these areas at youth levels are more likely to achieve professional status. Importantly, such technical competencies are not solely innate; they are also cultivated through structured and unstructured practice, reinforcing the relevance of developmental environments in nurturing talent [12,13]. However, technical skills alone are insufficient in a sport as dynamic and time sensitive as soccer. Players require game intelligence and must perceive, interpret, and act on information in real time, under pressure, and within ever-changing contexts. Perceptual-cognitive skills (PCS), which encompass anticipation and decision-making, thus, play a crucial role in high-level performance [14]. Anticipation involves the ability to predict events before they unfold, while decision-making involves selecting and executing contextually appropriate actions. Skilled players consistently outperform their less skilled peers on these dimensions [15–17] as well as associated with higher adult performance levels [18]. Such advantages are underpinned by efficient visual search strategies and enriched memory representations that facilitate rapid and effective responses [15,19].

Physical attributes are among the most frequently researched domains in TI and are often the first to be objectively measured through field-based tests [20]. Metrics such as sprint speed, agility, jumping ability, power, and endurance capacity are widely collected and analysed [21]. These measurements are typically interpreted together with chronological age and assessments of biological maturity to contextualise a player’s physical profile [22]. While these attributes can discriminate between levels of competition or selection status (e.g., [9,23,24]), they should be interpreted with caution. Physical development has a non-linear trajectory and may not align directly with a player’s broader soccer potential [4]. But, when integrated within a multi-disciplinary evaluation, physical data can meaningfully inform selection decisions.

In contrast to the extensive assessment of physical and technical domains, psychological predictors remain one of the most under-assessed components of TI. This is surprising, given the consensus among coaches and sports scientists that psychological traits such as resilience, motivation, coachability, focus, and emotional regulation are essential to development and long-term success [25,26]. These attributes are not merely supportive of other skill domains, they often determine whether a player can persist through the adversity, competitive pressure, and transitional phases of elite sport [25,27,28]. Yet, psychological profiling in TI remains relatively underdeveloped, largely due to the challenges of reliable assessment and interpretation [29]. Unlike sprint times or passing accuracy, psychological traits do not lend themselves to straightforward measurement, and many are context-sensitive or evolve over time. Therefore, despite their importance, they remain peripheral in many TI frameworks. This is compounded by the growing recognition that psychological attributes do not operate in a vacuum [30].

Social factors also recently attracted growing interest within TI research [31]. Aspects such as family support, quality of coaching relationships, access to informal play, and cultural values all contribute to shaping the developmental environment [28,32,33]. For instance, players with supportive families often have more consistent access to transport, training, and emotional encouragement, factors that have been repeatedly linked to sustained engagement and performance progression [34,35]. Moreover, research on birthplace effects underscore the impact of growing up in smaller towns or soccer-centric regions, where opportunities for deliberate play and early participation are more prevalent [26,36]. Furthermore, the relative age effect (RAE; the overrepresentation of players born earlier in the selection year) can bias perceptions of maturity, leadership, and confidence [37,38]. Similarly, athletes from socio-economically advantaged backgrounds may have greater access to developmental resources, which in turn cultivates traits like self-efficacy and achievement motivation [26]. Therefore, sociocultural factors can play an important but often overlooked role in TI; particularly when athletes are raised in communities with strong sport infrastructure or a prevailing soccer culture [39]. As such, attributes may be both shaped by and misinterpreted through these contextual filters, warranting more nuanced assessment approaches that account for environmental moderators. However, scouts and coaches do not universally consider these sociological predictors, and studies often focus narrowly on European male samples, limiting the generalisability of findings [33].

Another persistent gap in the literature lies in understanding how coaches perceive and weigh these various predictors. Whilst coach perceptions and subjective opinions have not been empirically validated against long-term performance outcomes, coaches are still central to TI, serving as both gatekeepers and architects of opportunity due to the importance of their opinions during decision-making [40]. Yet relatively few studies have explored their conceptual frameworks or selection rationale. Larkin and O’Connor [30] found that Australian coaches and scouts prioritised technical and tactical attributes above physical or sociological considerations when evaluating U13 players. These findings have since been echoed in other contexts, with psychological and technical skills often cited as more valuable than anthropometric or physiological factors [25,41]. Understandably, there is an age-related gradient in coach perceptions, where younger players (≤ 13 years of age) are more difficult to accurately identify as they are further away from the endpoint of talent development (i.e., professional status), and thus scouts place greater emphasis on older athletes with more observable performance markers [42,43]. However, adding to the complexity of TI is the conceptual ambiguity surrounding commonly used constructs such as “game intelligence” and “technique” [44,45]. While widely referenced in both research and practice, these terms often lack precise and standardised definitions that can lead to variability in practitioner interpretations [46]. For example, ‘game intelligence’ can include perceptual-cognitive skills (e.g., anticipation, decision-making; [47]) as well as broader tactical understanding, while ‘technique’ may refer narrowly to soccer-specific motor skills (e.g., passing, shooting), or more holistically to technical-tactical execution under pressure [45]. Such conceptual ambiguity poses challenges for consistency and quality within and across TI programs [46].

Despite emerging insights, the current knowledge base remains limited in scope, especially with respect to female players and non-European contexts. The United States (U.S.) presents a unique landscape for TI, particularly due to the role of the collegiate system as a pathway to professional soccer. With approximately 4.5 million youth players across sexes [48,49], the college stage serves as a crucial intermediary between youth and senior soccer [50]. However, the TI process in this context is shaped by the National Collegiate Athletic Association (NCAA) rules that limits recruitment activity until age 15 or 16 years, a model that contrasts significantly with the early academy pathways seen in Europe and elsewhere [51,52]. College coaches, operating independently from professional clubs, must often recruit athletes who are already ‘performance ready’, thereby narrowing their focus to observable attributes and outcomes [53,54]. Given the constraints of the TI processes of college soccer in the U.S. compared with the typical talent pathways of other female and/or male soccer players across the globe such as in South America and Europe [51,52]., it makes it difficult to draw upon literature from other contexts (e.g., academy-based systems), thus highlighting the need for further investigation into TI processes specifically in college soccer in the US. Understanding the attributes college coaches prioritise, and how their perceptions may vary by sex or program type, is also essential for aligning TD efforts with recruitment realities. Furthermore, the U.S. Soccer federation has recently launched a committee with the NCAA to provide recommendations for the future of men’s and women’s college soccer programs and their connectivity to the professional game, making any research in TI timely and with applied implications. Thus, in the present study, we examined what college soccer coaches in the U.S. consider as important for informing their TI decisions, and whether these perceptions change according to working within male or female programs.

## Methods

### Participants

We recruited professional soccer coaches from Divisions I (DI) and II (DII) of the NCAA. These coaches are responsible for both identifying and recruiting players for their college soccer teams as well as planning, delivering, and evaluating practice. Thus, henceforth they will be referred to simply as “coaches”. Given the coaches’ prominent roles in such processes, and the limited timeframe between recruitment to an NCAA program (age 16+) and the need for athlete’s to be “performance ready” (age 18+), coach perceptions were deemed a valid focus of TI in the context of the current study.These coaches were approached through email (collated via open-access team websites; e.g., www.ncaa.com)), containing the rationale of the study, instructions on how to complete the survey, as well as contact details should they require any further details. Snowball sampling was used to increase visibility, where participants were encouraged to circulate to their networks and peers [55]. To meet the inclusion criteria, coaches were required to be ≥ 18 years of age and working (full- or part-time) in the coaching department in either a DI or DII women’s or men’s soccer program. There was no condition on how much previous experience participants had in these roles. All participants were provided with a participant information sheet on the first page of the questionnaire and implied consent was given on submission. The procedure was in line with the Declaration of Helsinki and approved by a university research ethics committee (23/SPS/047). From the 2,630 coaches approached across all programs and divisions, a total of 178 coaches responded (6.8% response rate) and completed the survey, which is comparable to similar studies (e.g., [43]). Coaches had a mean chronological age of 39.2 ± 12.0 years, and 14.5 ± 10.4 years’ experience in a coaching role, with 13.2 ± 10.2 years of this being in college soccer. Among participants, 77 (43.3%) were head coaches, 15 (8.4%) were associate coaches, 82 (46.1%) were assistant coaches, and 4 (2.2%) were classified as ‘other’ such graduate coach and/or recruiting coordinator. A total of 71 (60.1%) identified as female and 107 (39.9%) as male. A breakdown of participant characteristics can be seen in Table 1. containing the rationale of the study, instructions on how to complete the survey, as well as contact details should they require any further details. Snowball sampling was used to increase visibility, where participants were encouraged to circulate to their networks and peers [55]. To meet the inclusion criteria, coaches were required to be ≥ 18 years of age and working (full- or part-time) in the coaching department in either a DI or DII women’s or men’s soccer program. There was no condition on how much previous experience participants had in these roles. All participants were provided with a participant information sheet on the first page of the questionnaire and implied consent was given on submission. The procedure was in line with the Declaration of Helsinki and approved by a university research ethics committee (23/SPS/047). From the 2,630 coaches approached across all programs and divisions, a total of 178 coaches responded (6.8% response rate) and completed the survey with the total number of respondents, comparable to similar studies (e.g., [43]). Coaches had a mean chronological age of 39.2 ± 12.0 years, and 14.5 ± 10.4 years’ experience in a coaching role, with 13.2 ± 10.2 years of this being in college soccer. Among participants, 77 (43.3%) were head coaches, 15 (8.4%) were associate coaches, 82 (46.1%) were assistant coaches, and 4 (2.2%) were classified as ‘other’ such as a recruiting coordinator. A total of 71 (60.1%) identified as female and 107 (39.9%) as male. A breakdown of participant characteristics can be seen in Table 1.

**Table 1:** Participant characteristics (%).

		Women’s Soccer		Men’s Soccer	
		DI	DII	DI	DII
N		62 (53.4)	54 (46.6)	33 (53.2)	29 (46.8)
Age (years)		41.8 ± 12.4	35.6 ± 12.0	39.6 ± 9.2	40.1 ± 12.7
	Male	20 (32.3)	25 (46.3)	33 (53.2)	29 (46.8)
	Female	42 (67.7)	29 (53.7)	0 (0)	0 (0)
Coaching role	Head Coach	24 (38.7)	20 (37.0)	17 (51.5)	16 (55.2)
	Associate Coach	10 (16.1)	1 (1.9)	4 (12.1)	0 (0)
	Assistant Coach	28 (45.2)	29 (53.7)	12 (36.4)	13 (44.8)
	Other	0 (0)	4 (7.4)	0 (0)	0 (0)
Scouting experience (years)		16.2 ± 10.8	11.9 ± 10.9	15.5 ± 8.8	14.4 ± 9.6
College specific experience (years)		15.4 ± 10.9	9.9 ± 9.6	14.7 ± 9.2	13.1 ± 9.8
Scouting events attend (previous season)		8.4 ± 5.3	7.0 ± 4.4	6.8 ± 3.9	6.4 ± 3.6

### Survey

An adapted digital survey that has previously been used to examine perceptions of talent in youth soccer players was used [23]. The survey was refined based on updated research (e.g., [41–43,56]) and for terminology by the lead researcher who was acquainted with the youth soccer environment in the U.S. The survey was pilot tested by a youth soccer coach for estimated time to completion and help improve terminology consistency and clarity. In total, the survey contained 119 questions. In section one, coaches were asked about their background (e.g., “*how many years have you been scouting in soccer?*”). In section two, coaches were asked to rate the importance of attributes (e.g., playing experience) and methods (e.g., live games) in TI. In the final section, coaches were asked their perceived importance of individual skills/attributes when identifying players, which were categorised into physical (e.g., agility), psychological (e.g., grit), technical (e.g., passing), game intelligence (e.g., decision-making), social (e.g., teamwork), and other (e.g., birthdate), as per Roberts et al. [25]. Coaches rated each individual attribute within the category using a numerical 5-point Likert scale. All points were labelled with verbal anchors presented as follows: 1 = Not at All Important; 2 = Not Important; 3 = Important; 4 = Very Important; 5 = Extremely Important. Given the symmetrical nature of the scale, the distances between scales points were assumed to be equidistant, and all responses were treated as interval-level data for analysis [57]. Additionally, using multiple-choice responses, coaches were asked what they perceived as the most, second, and third most important skill for each category. The survey opened during separate soccer seasons, with the female soccer coaches on August 18^th^, 2023, and December 3^rd^, 2024, for the male soccer coaches. Both surveys were open for approximately 12 weeks. Email promotions were used every four-weeks to encourage completion.

### Data analysis

Responses from the survey were exported into Microsoft Excel (Microsoft Corp., Redmond, WA, United States) and subsequently R Statistical Software (v4.4.2; R Core Team 2021) for further analysis. Prior to the main analysis, preliminary screening of the data revealed violations in assumptions of normality (skewness), homogeneity of (co)variance, and presence of outliers within the data. Therefore, statistical methods were employed that can provide accurate results when traditional data assumptions are not met [52]. Descriptive statistics were calculated as trimmed means (20%) and 95% conﬁdence intervals derived from 1000 bootstrapped samples as robust estimators of the mean importance of each variable within the survey, for both male and female programs [58]. In addition, inter-rater agreement was calculated through the *r*_wg_ statistic, which indicates observed disagreement as a proportion of theoretical chance disagreement [59,60]. A higher agreement score indicates that aggregated individual responses to each survey item accurately reflects the collective perspective of the coaches [61]. Inter-rater agreement statistics were interpreted using the following recommended standards [62]: 0–0.30 (lack of), 0.31–0.50 (weak), 0.51–0.70 (moderate), 0.71–0.90 (strong), and 0.91–1.0 (very strong). Further, robust independent samples *t*-tests [63] were used to assess for any potential differences in coaches a working within the female and male programs. This analysis again utilised trimmed means (20%) and 95% conﬁdence intervals derived from 1000 bootstrapped samples. Data were considered significant if *p* < 0.05 and if the 95% confidence interval of the difference in trimmed means (*M*_diff_) did not cross zero. Explanatory measures of effect size (ξ [xi]; [64]) were calculated and interpreted as 0.10 = small, 0.30 = medium, and 0.50 = large [65]. All follow-up analysis were performed using the yuenbt () function from the WRS2 package [66]. Finally, frequency analysis was conducted to demonstrate the proportion of respondents ranking their first, second, and third rated most important variables.

## Results

### Perceptions of attributes for coaches and methods used during talent identification

College-specific soccer knowledge was perceived as the most important attribute for soccer coaches to possess during TI with the highest mean rating of importance for coaches from both female (M = 4.6, 95% CI [4.4, 4.7]) and male (M = 4.5, 95% CI [4.3, 4.7]) programs. Additionally, college-specific soccer knowledge showed strong (*r*_wg_= 0.76) and moderate (*r*_wg_= 0.69) inter-rater agreement in female and male soccer, respectively (Table 2). Frequency analysis of the top three ranked most important attributes confirmed the previous findings with most coaches in female (55.2%) and male programs (64.5%), ranking college soccer specific knowledge as the most important attribute for TI purposes. Further analysis revealed a small (ξ = 0.27) but significant difference between coaches working within the female game, who perceived prior coaching experience as significantly more important than coaches working in the male game (t = 2.87, p < 0.001, *M*_diff_ = 0.5, 95% CI [0.1, 0.8]).

**Table 2:** Estimated trim mean (± SD) of men’s and women’s program responses to the Likert scale attributes and methods of scouting, with 95% confidence intervals, within group agreement (*r*_*wg*_ (Int)), as well as along with frequency (%) of most, second most and third most important attribute.

	Women’s Program						Men’s Program					
	ETM	95% CI	*r*_wg_ (Int)	1st	2^nd^	3rd	ETM	95% CI	*r*_wg_ (Int)	1st	2nd	3rd
Attribute												
College specific knowledge	4.6 ± 0.7 **EI**	[4.4, 4.7]	0.76 **SA**	64 (55.2)	25 (21.6)	18 (15.5)	4.5 ± 0.8 **EI**	[4.3, 4.7]	0.69 **MA**	40 (64.5)	9 (14.5)	9 (14.5)
Coaching experience***	4.2 ± 0.9 **VI**	[4.0, 4.4]	0.56 **MA**	20 (17.2)	45 (38.8)	37 (31.9)	3.7 ± 0.9 **VI**	[3.5, 4.0]	0.63 **MA**	8 (12.9)	22 (35.5)	23 (37.1)
Scouting experience	4.1 ± 0.9 **VI**	[3.9, 4.3]	0.62 **MA**	26 (22.4)	31 (26.7)	34 (29.3)	3.7 ± 0.9 **VI**	[3.5, 4.1]	0.46 **WA**	11 (17.7)	19 (30.6)	18 (29.0)
Playing experience	3.3 ± 0.9 **I**	[3.1, 3.4]	0.58 **MA**	6 (5.2)	12 (10.3)	19 (16.4)	3.3 ± 0.9 **I**	[2.9, 3.5]	0.26 **LoA**	3 (4.8)	10 (16.1)	10 (16.1)
Formal education	2.9 ± 1.0 **I**	[2.7, 3.1]	0.52 **MA**	–	3 (2.6)	8 (6.9)	3.0 ± 1.0 **I**	[2.7, 3.3]	0.42 **WA**	–	2 (3.2)	2 (3.2)
Methods												
Games (live)	5.0 ± 0.6 **EI**	[4.8, 5.0]	0.82 **SA**	108 (93.1)	3 (2.6)	3 (2.6)	4.8 ± 0.5 **EI**	[4.7, 5.0]	0.85 **SA**	55 (88.7)	2 (3.2)	3 (4.8)
Games (video)*	3.5 ± 0.7 **VI**	[3.3, 3.6]	0.75 **SA**	3 (2.6)	67 (57.8)	20 (17.2)	3.8 ± 0.7 **VI**	[3.5, 4.0]	0.76 **SA**	4 (6.5)	31 (50.0)	12 (19.4)
Video highlights	3.2 ± 0.7 **I**	[3.1, 3.4]	0.77 **SA**	5 (4.3)	17 (14.7)	45 (38.8)	3.4 ± 0.7 **I**	[3.2, 3.7]	0.72 **SA**	1 (1.6)	18 (29.0)	17 (27.4)
View of other coaches	3.1 ± 0.9 **I**	[2.8, 3.3]	0.56 **MA**	–	19 (16.4)	23 (19.8)	3.2 ± 0.9 **I**	[2.8, 3.4]	0.54 **MA**	2 (3.2)	8 (12.9)	14 (22.6)
Statistics	2.7 ± 0.8 **I**	[2.5, 2.8]	0.69 **MA**	–	7 (6.0)	13 (11.2)	2.9 ± 0.8 **I**	[2.7, 3.1]	0.65 **MA**	–	3 (4.8)	13 (21.0)
Testing	2.7 ± 0.8 **I**	[2.5, 2.8]	0.66 **MA**	–	3 (2.6)	11 (94.5)	2.6 ± 0.7 **I**	[2.4, 2.8]	0.74 **SA**	–	–	3 (4.8)
Social media clips	2.1 ± 0.6 **NI**	[2.0, 2.3]	0.80 **SA**	–	–	1 (0.9)	1.8 ± 0.9 **NI**	[1.6, 2.1]	0.63 **MA**	–	–	–

ETM = estimated trim mean; EI = extremely important; VI = very important; I = important; NVI = not very important; NIA = not important at all); (95% CI), and within group agreement (interpretation (SA = strong agreement; MA = moderate agreement; WA = weak agreement; LoA = lack of agreement).

Watching games (live) was perceived as the most important method for coaches in both the female (M = 5.0, 95% CI [4.8, 5.0]) and male (M = 4.8, 95% CI [4.7, 5.0]) programs. The proportion of coach’s rankings confirmed this finding, with 93.1% and 88.7% of coach’s working within the female and male programs, respectively, ranking live games as the most important method for TI (Table 2). All ratings of methods used demonstrated moderate to strong agreement (*r*_wg _≥ 0.54 and ≤ 0.85). Small but significant differences were found in methods used by coaches for TI. Coaches in female soccer perceived games (video) as less important (t = −2.20, p < 0.05, Mdiff = 0.3, 95% CI [−0.6, −0.0], ξ = 0.25) and social media clips as more important (t = 1.99, p < 0.05, Mdiff = 0.4, 95% CI [0.0, 0.7], ξ = 0.24) than those in male soccer.

### Perceptions of attributes

Pace (female program: M = 4.4, 95% CI [4.2, 3.5]; male program: M = 4.1, 95% CI [3.9, 4.2]) and stamina (female program: M = 4.2, 95% CI [4.0, 4.4]; male program: M = 4.2, 95% CI [4.0, 4.4]) were rated as the most important physical attribute coaches considered when scouting players, with pace further demonstrating its perceived importance through being ranked as the most important attribute to consider in both female (31.0%) and male (33.9%) programs. All attributes, except for jumping reach for coaches working within the male program, demonstrated strong inter-rater agreement (*r*_wg _≥ 0.71 and ≤ 0.80; Table 3). Coaches and scouts within the female program perceived pace (t = 2.64, p < 0.05, Mdiff = 0.3, 95% CI [0.1, 0.6], ξ = 0.29), agility (t = 3.56, p < 0.01, Mdiff = 0.5, 95% CI [0.2, 0.7], ξ = 0.33), and acceleration (t = 3.18, p < 0.01, Mdiff = 0.4, 95% CI [0.1, 0.6], ξ = 0.37) as significantly more important than in the male program, with moderate effect sizes.

**Table 3:** Estimated trim mean Mean (± SD) of men’s and women’s program responses to the Likert scale importance for of players attributes, with 95% confidence intervals, within group agreement (*r*_*wg*_ (Int)), as well as along with frequency (%) of most, second most and third most important attribute.

	Women’s Program						Men’s Program					
Attribute	ETM	95% CI	*r*_wg_ (Int)	1st	2nd	3rd	ETM	95% CI	*r*_wg_ (Int)	1^st^	2nd	3rd
***Physical***												
Pace*	4.4 ± 0.7 **VI**	[4.2, 4.5]	0.79 **SA**	36 (31.0)	27 (23.3)	16 (13.8)	4.1 ± 0.7 **VI**	[3.9, 4.2]	0.79 **SA**	21 (33.9)	16 (25.8)	8 (12.9)
Acceleration**	4.3 ± 0.7 **VI**	[4.2, 4.4]	0.74 **SA**	27 (23.3)	28 (24.1)	12 (10.3)	3.9 ± 0.7 **VI**	[3.7, 4.1]	0.78 **SA**	3 (4.8)	7 (11.3)	11 (17.7)
Stamina	4.2 ± 0.7 **VI**	[4.0, 4.4]	0.74 **SA**	12 (10.3)	13 (11.2)	25 (21.6)	4.2 ± 0.7 **VI**	[4.0, 4.4]	0.78 **SA**	13 (21.0)	16 (25.8)	11 (17.7)
Agility**	4.2 ± 0.7 **VI**	[4.0, 4.3]	0.76 **SA**	14 (12.1)	18 (15.5)	5 (8.1)	3.8 ± 0.6 **VI**	[3.6, 3.9]	0.80 **SA**	5 (8.1)	6 (9.7)	7 (11.3)
Balance	3.9 ± 0.7 **VI**	[3.7, 4.0]	0.72 **SA**	5 (4.3)	7 (6.0)	4 (3.4)	3.8 ± 0.7 **VI**	[3.6, 4.0]	0.77 **SA**	4 (6.5)	3 (4.8)	10 (16.1)
Natural fitness	3.8 ± 0.8 **VI**	[3.6, 4.0]	0.72 **SA**	20 (17.2)	6 (5.2)	12 (10.3)	3.8 ± 0.8 **VI**	[3.6, 4.0]	0.71 **SA**	14 (22.6)	4 (6.5)	7 (11.3)
Strength	3.6 ± 0.7 **VI**	[4.5, 3.8]	0.74 **SA**	2 (1.7)	16 (13.8)	18 (15.5)	3.7 ± 0.7 **VI**	[3.5, 3.9]	0.77 **SA**	2 (3.2)	10 (16.1)	8 (12.9)
Jumping reach	2.9 ± 0.8 **I**	[2.8, 3.1]	0.71 **SA**	–	1 (0.9)	–	3.0 ± 0.9 **I**	[2.7, 3.3]	0.64 **MA**	–	–	–
***Psychological***												
Work rate	5.0 ± 0.5 **EI**	[4.9, 5.0]	0.88 **SA**	20 (17.2)	18 (15.5)	17 (14.7)	4.9 ± 0.6 **EI**	[4.7, 5.0]	0.84 **SA**	10 (16.1)	11 (17.7)	8 (12.9)
Coachability	4.9 ± 0.5 **EI**	[4.8, 5.0]	0.87 **SA**	35 (30.2)	18 (15.5)	16 (13.8)	4.8 ± 0.6 **EI**	[4.6, 5.0]	0.80 **SA**	20 (32.3)	11 (17.7)	8 (12.9)
Confidence	4.7 ± 0.7 **EI**	[4.6, 4.9]	0.84 **SA**	8 (6.9)	12 (10.3)	6 (5.2)	4.8 ± 0.7 **EI**	[4.6, 4.9]	0.79 **SA**	2 (3.2)	4 (6.5)	1 (1.6)
Winning mindset	4.6 ± 0.7 **EI**	[4.4, 4.7]	0.76 **SA**	12 (10.3)	4 (3.4)	10 (8.6)	4.6 ± 0.7 **EI**	[4.4, 4.8]	0.79 **SA**	1 (1.6)	5 (8.1)	8 (12.9)
Resilient	4.6 ± 0.7 **EI**	[4.4, 4.7]	0.77 **SA**	5 (4.3)	18 (15.5)	12 (10.3)	4.5 ± 0.7 **EI**	[4.3, 4.7]	0.74 **SA**	3 (4.8)	4 (6.5)	2 (3.2)
Determination	4.5 ± 0.7 **EI**	[4.3, 4.6]	0.75 **SA**	6 (5.2)	5 (4.3)	5 (4.3)	4.5 ± 0.7 **EI**	[4.3, 4.7]	0.79 **SA**	2 (3.2)	3 (4.8)	3 (4.8)
Grit	4.4 ± 0.7 **VI**	[4.3, 4.6]	0.75 **SA**	5 (4.3)	8 (6.9)	10 (8.6)	4.5 ± 0.8 **EI**	[4.3, 4.8]	0.70 **SA**	9 (14.5)	7 (11.3)	2 (3.2)
Motivation	4.4 ± 0.7 **VI**	[4.3, 4.6]	0.78 **SA**	4 (3.4)	9 (7.8)	4 (3.4)	4.3 ± 0.7 **VI**	[4.0, 4.5]	0.74 **SA**	1 (1.6)	2 (3.2)	4 (6.5)
Commitment	4.3 ± 0.6 **VI**	[4.2, 4.4]	0.77 **SA**	9 (7.8)	13 (11.2)	12 (10.3)	4.4 ± 0.7 **VI**	[4.1, 4.6]	0.71 **SA**	6 (9.7)	2 (3.2)	4 (6.5)
Concentration	4.3 ± 0.7 **VI**	[4.1, 4.4]	0.74 **SA**	1 (0.9)	2 (1.7)	1 (0.9)	4.3 ± 0.7 **VI**	[4.1, 4.5]	0.74 **SA**	–	2 (3.2)	2 (3.2)
Positive mindset	4.3 ± 0.7 **VI**	[4.1, 4.5]	0.73 **SA**	–	3 (2.6)	2 (1.7)	4.3 ± 0.7 **VI**	[4.1, 4.5]	0.77 **SA**	3 (4.8)	1 (1.6)	9 (14.5)
Composure	4.2 ± 0.7 **VI**	[4.0, 4.3]	0.75 **SA**	5 (4.3)	7 (6.0)	6 (5.2)	4.4 ± 0.8 **VI**	[4.2, 4.6]	0.77 **SA**	1 (1.6)	2 (3.2)	1 (1.6)
Personality	4.1 ± 0.9 **VI**	[3.9, 4.3]	0.64 **MA**	1 (0.9)	4 (3.4)	3 (2.6)	4.2 ± 0.8 **VI**	[3.9, 4.5]	0.66 **MA**	–	5 (8.1)	3 (4.8)
Bravery	4.1 ± 0.7 **VI**	[3.9, 4.2]	0.74 **SA**	2 (1.7)	4 (3.4)	4 (3.4)	4.0 ± 0.7 **VI**	[3.8, 4.2]	0.76 **SA**	3 (4.8)	1 (1.6)	3 (4.8)
Goal-orientated	3.9 ± 0.8 **VI**	[3.7, 4.1]	0.71 **SA**	1 (0.9)	1 (0.9)	–	3.9 ± 0.8 **VI**	[3.6, 4.2]	0.69 **MA**	–	1 (1.6)	1 (1.6)
Leadership	3.7 ± 0.8 **VI**	[3.6, 4.0]	0.64 **MA**	1 (0.9)	3 (2.6)	4 (3.4)	3.8 ± 0.8 **VI**	[3.6, 4.1]	0.69 **MA**	–	1 (1.6)	3 (4.8)
Aggression	3.7 ± 0.8 **VI**	[3.5, 3.9]	0.69 **MA**	1 (0.9)	1 (0.9)	4 (3.4)	3.5 ± 0.8 **VI**	[3.3, 3.8]	0.69 **MA**	1 (1.6)	1 (1.6)	–
***Technical***												
Ball control	4.6 ± 0.7 **EI**	[4.5, 4.8]	0.77 **SA**	50 (43.1)	23 (19.8)	12 (10.3)	4.5 ± 0.6 **EI**	[4.3, 4.7]	0.79 **SA**	25 (40.3)	16 (25.8)	2 (3.2)
Technique	4.5 ± 0.7 **EI**	[4.3, 4.6]	0.74 **SA**	26 (22.4)	12 (10.3)	10 (8.6)	4.5 ± 0.7 **EI**	[4.3, 4.7]	0.78 **SA**	26 (41.9)	10 (16.1)	9 (14.5)
Receiving the ball	4.5 ± 0.7 **EI**	[4.4, 4.6]	0.74 **SA**	9 (7.8)	14 (12.1)	11 (9.5)	4.4 ± 0.7 **VI**	[4.1, 4.6]	0.74 **SA**	4 (6.5)	10 (16.1)	11 (17.7)
Passing (short)	4.4 ± 0.8 **VI**	[4.2, 4.6]	0.71 **SA**	6 (5.2)	19 (16.4)	20 (17.2)	4.4 ± 0.7 **VI**	[4.2, 4.6]	0.75 **SA**	2 (3.2)	13 (21.0)	17 (27.4)
Finishing	4.4 ± 0.7 **VI**	[4.2, 4.5]	0.77 **SA**	9 (7.8)	10 (8.6)	9 (7.8)	4.0 ± 0.7 **VI**	[3.9, 4.2]	0.79 **SA**	1 (1.6)	–	8 (12.9)
Passing (long)	4.2 ± 0.8 **VI**	[4.0, 4.4]	0.69 **SA**	1 (0.9)	6 (5.2)	12 (10.3)	4.0 ± 0.6 **VI**	[3.8, 4.2]	0.80 **SA**	–	1 (1.6)	1 (1.6)
Running with the ball	4.0 ± 0.8 **VI**	[3.7, 4.2]	0.68 **SA**	2 (1.7)	6 (5.2)	9 (7.8)	3.9 ± 0.7 **VI**	[3.7, 4.0]	0.79 **SA**	–	2 (3.2)	1 (1.6)
Shooting	4.0 ± 0.7 **VI**	[3.8, 4.1]	0.74 **SA**	–	1 (0.9)	3 (2.6)	3.8 ± 0.7 **VI**	[3.6, 4.0]	0.79 **SA**	1 (1.6)	–	2 (3.2)
Tackling	4.0 ± 0.8 **VI**	[3.7, 4.2]	0.71 **SA**	–	3 (2.6)	6 (5.2)	3.8 ± 0.7 **VI**	[3.5, 4.0]	0.75 **SA**	–	1 (1.6)	4 (6.5)
Chance creation	3.8 ± 0.8 **VI**	[3.6, 4.1]	0.72 **SA**	7 (6.0)	12 (10.3)	5 (4.3)	3.8 ± 0.7 **VI**	[3.7, 4.0]	0.75 **SA**	1 (1.6)	4 (6.5)	4 (6.5)
Dribbling	3.7 ± 0.7 **VI**	[3.6, 4.0]	0.73 **SA**	3 (2.6)	4 (3.4)	6 (5.2)	3.7 ± 0.7 **VI**	[3.5, 3.9]	0.73 **SA**	1 (1.6)	3 (4.8)	–
Heading	3.6 ± 0.8 **VI**	[3.5, 3.9]	0.69 **MA**	2 (1.7)	2 (1.7)	8 (6.9)	3.6 ± 0.8 **VI**	[3.4, 3.8]	0.73 **SA**	1 (1.6)	2 (3.2)	1 (1.6)
Marking	3.5 ± 0.8 **VI**	[3.4, 3.8]	0.64 **MA**	–	3 (2.6)	2 (1.7)	3.6 ± 0.8 **VI**	[3.4, 3.8]	0.71 **SA**	–	–	2 (3.2)
Crossing	3.4 ± 0.7 **I**	[3.2, 3.5]	0.74 **SA**	–	–	1 (0.9)	3.5 ± 0.7 **VI**	[3.3, 3.7]	0.77 **SA**	–	–	–
Interceptions	3.3 ± 0.7 **I**	[3.2, 3.5]	0.76 **SA**	–	1 (0.9)	–	3.6 ± 0.7 **VI**	[3.4, 3.8]	0.79 **SA**	–	–	–
Blocking	3.2 ± 0.8 **I**	[3.1, 3.3]	0.72 **SA**	1 (0.9)	–	2 (1.7)	3.4 ± 0.9 **I**	[3.1, 3.3]	0.61 **MA**	–	–	–
***Game Intelligence***												
Decision-making	4.8 ± 0.6 **EI**	[4.6, 4.9]	0.85 **SA**	64 (55.2)	27 (23.3)	16 (13.8)	4.6 ± 0.7 **EI**	[4.4, 4.8]	0.77 **SA**	34 (54.8)	13 (21.0)	8 (12.9)
Information processing	4.4 ± 0.7 **VI**	[4.3, 4.6]	0.76 **SA**	11 (9.5)	16 (13.8)	15 (12.9)	4.4 ± 0.8 **VI**	[4.2, 4.6]	0.68 **SA**	7 (11.3)	16 (25.8)	7 (11.3)
Positional understanding	4.4 ± 0.7 **VI**	[4.2, 4.5]	0.73 **SA**	7 (6.0)	17 (14.7)	20 (17.2)	4.4 ± 0.7 **VI**	[4.1, 4.6]	0.76 **SA**	4 (6.5)	13 (21.0)	10 (16.1)
Anticipation	4.3 ± 0.7 **VI**	[4.1, 4.5]	0.72 **SA**	8 (6.9)	13 (11.2)	15 (12.9)	4.2 ± 0.7 **VI**	[4.0, 4.4]	0.78 **SA**	5 (8.1)	6 (9.7)	8 (12.9)
Tactical knowledge	4.3 ± 0.8 **VI**	[4.1, 4.5]	0.70 **SA**	8 (6.9)	13 (11.2)	18 (15.5)	4.1 ± 0.7 **VI**	[3.9, 4.3]	0.72 **SA**	5 (8.1)	4 (6.5)	17 (27.4)
Creativity	4.0 ± 0.8 **VI**	[3.7, 4.2]	0.67 **MA**	2 (1.7)	13 (11.2)	17 (14.7)	3.9 ± 0.8 **VI**	[3.7, 4.3]	0.66 **MA**	3 (4.8)	5 (8.1)	8 (12.9)
Visual search	4.0 ± 0.8 **VI**	[3.8, 4.3]	0.67 **MA**	8 (6.9)	5 (4.3)	6 (5.2)	3.9 ± 0.9 **VI**	[3.6, 4.2]	0.62 **MA**	2 (3.2)	2 (3.2)	2 (3.2)
Cue utilisation	4.0 ± **VI**	[3.7, 4.2]	0.70 **SA**	8 (6.9)	12 (10.3)	9 (7.8)	3.8 ± 0.8 **VI**	[3.6, 4.1]	0.65 **MA**	2 (3.2)	3 (4.8)	2 (3.2)
***Social***												
Teamwork	4.8 ± 0.6 **EI**	[4.7, 4.9]	0.81 **SA**	18 (15.5)	18 (15.5)	21 (18.1)	4.7 ± 0.6 **EI**	[4.5, 4.8]	0.80 **SA**	12 (19.4)	12 (19.4)	11 (17.7)
Communication	4.6 ± 0.7 **EI**	[4.5, 4.8]	0.77 **SA**	15 (12.9)	26 (22.4)	16 (13.8)	4.3 ± 0.6 **VI**	[4.1, 4.5]	0.80 **SA**	8 (12.9)	5 (8.1)	9 (14.5)
Responsible	4.5 ± 0.6 **EI**	[4.4, 4.7]	0.79 **SA**	12 (10.3)	11 (9.5)	17 (14.7)	4.5 ± 0.6 **EI**	[4.3, 4.7]	0.79 **SA**	6 (9.7)	12 (19.4)	8 (12.9)
Adaptability	4.5 ± 0.6 **EI**	[4.3, 4.6]	0.79 **SA**	22 (19.0)	9 (7.8)	11 (9.5)	4.4 ± 0.7 **VI**	[4.2, 4.6]	0.72 **SA**	11 (17.7)	10 (16.1)	4 (6.5)
Builds relationships	4.5 ± 0.7 **EI**	[4.3, 4.6]	0.73 **SA**	21 (18.1)	10 (8.6)	7 (6.0)	4.0 ± 0.7 **VI**	[3.8, 4.2]	0.75 **SA**	4 (6.5)	5 (8.1)	6 (9.7)
Accepts criticism	4.4 ± 0.8 **VI**	[4.2, 4.5]	0.70 **SA**	12 (10.3)	22 (19.0)	9 (7.8)	4.3 ± 0.7 **VI**	[4.0, 4.5]	0.73 **SA**	6 (9.7)	7 (11.3)	5 (8.1)
Lifestyle management	4.3 ± 0.8 **VI**	[4.1, 4.5]	0.72 **SA**	4 (3.4)	9 (7.8)	15 (12.9)	4.3 ± 0.7 **VI**	[4.1, 4.5]	0.76 **SA**	9 (14.5)	6 (9.7)	5 (8.1)
Prepared	4.3 ± 0.7 **VI**	[4.1, 4.4]	0.76 **SA**	5 (4.3)	2 (1.7)	10 (8.6)	4.1 ± 0.6 **VI**	[3.9, 4.3]	0.79 **SA**	3 (4.8)	2 (3.2)	4 (6.5)
Ability to excel	3.8 ± 0.7 **VI**	[3.7, 4.0]	0.72 **SA**	3 (2.6)	3 (2.6)	4 (3.4)	4.0 ± 0.8 **VI**	[3.7, 4.2]	0.67 **MA**	2 (3.2)	–	5 (8.1)
Role model	3.7 ± 0.8 **VI**	[3.5, 3.9]	0.67 **MA**	1 (0.9)	2 (1.7)	2 (1.7)	3.7 ± 0.7 **VI**	[3.5, 3.9]	0.75 **SA**	–	–	2 (3.2)
Independence	3.6 ± 0.8 **VI**	[3.5, 3.8]	0.68 **MA**	1 (0.9)	1 (0.9)	2 (1.7)	3.7 ± 0.8 **VI**	[3.4, 3.9]	0.68 **MA**	–	2 (3.2)	–
Assertive	3.6 ± 0.8 **VI**	[3.5, 3.7]	0.69 **MA**	1 (0.9)	2 (1.7)	2 (1.7)	3.6 ± 0.8 **VI**	[3.4, 3.8]	0.70 **SA**	1 (1.6)	–	1 (1.6)
Not easily intimidated	3.5 ± 0.8 **VI**	[3.4, 3.7]	0.68 **MA**	1 (0.9)	1 (0.9)	–	3.7 ± 0.7 **VI**	[3.5, 4.0]	0.74 **SA**	–	1 (1.6)	2 (3.2)
***Other***												
Innate talent	3.6 ± 0.8 **VI**	[3.4, 3.7]	0.65 **MA**	36 (31.0)	25 (21.6)	19 (16.4)	3.4 ± 0.8 **I**	[3.2, 3.6]	0.65 **MA**	17 (27.4)	10 (16.1)	10 (16.1)
Lifestyle	3.5 ± 0.9 **VI**	[3.4, 3.7]	0.62 **MA**	24 (20.7)	18 (15.5)	20 (17.2)	3.7 ± 0.9 **VI**	[3.5, 4.0]	0.62 **MA**	15 (24.2)	11 (17.7)	12 (19.4)
X-factor	3.4 ± 1.1 **I**	[3.2, 3.5]	0.39 **WA**	17 (14.7)	12 (10.3)	18 (15.5)	3.4 ± 1.0 **I**	[3.1, 3.5]	0.49 **WA**	2 (3.2)	9 (14.5)	12 (19.4)
Support from family	3.2 ± 1.0 **I**	[3.0, 3.4]	0.50 **MA**	18 (15.5)	15 (12.9)	19 (16.4)	3.0 ± 1.0 **I**	[2.7, 3.3]	0.53 **MA**	5 (8.1)	8 (12.9)	8 (12.9)
Education level	2.9 ± 0.9 **I**	[2.8, 3.1]	0.62 **MA**	4 (3.4)	12 (10.3)	12 (10.3)	3.2 ± 1.0 **I**	[3.0, 3.4]	0.55 **MA**	14 (22.6)	6 (9.7)	4 (6.5)
Biological maturation	2.9 ± 0.8 **I**	[2.8, 3.0]	0.65 **MA**	2 (1.7)	8 (6.9)	6 (5.2)	2.9 ± 0.7 **I**	[2.7, 3.2]	0.73 **SA**	2 (3.2)	5 (8.1)	6 (9.7)
Current soccer club/coach	2.7 ± 0.9 **I**	[2.6, 2.9]	0.57 **MA**	8 (6.9)	17 (14.7)	12 (10.3)	2.8 ± 1.0 **I**	[2.6, 3.2]	0.52 **SA**	3 (4.8)	7 (11.3)	4 (6.5)
Left/right foot dominant	2.5 ± 1.0 **I**	[2.3, 2.8]	0.51 **SA**	6 (5.2)	5 (4.3)	4 (3.4)	2.4 ± 1.0 **NV**	[2.2, 2.6]	0.54 **SA**	–	4 (6.5)	2 (3.2)
Appearance	2.0 ± 0.9 **NV**	[1.8, 2.3]	0.58 **MA**	1 (0.9)	2 (1.7)	3 (2.6)	2.2 ± 1.0 **NV**	[1.9, 2.5]	0.46 **WA**	1 (1.6)	1 (1.6)	2 (3.2)
Cultural background	1.8 ± 0.9 **NV**	[1.6, 2.0]	0.67 **MA**	–	1 (0.9)	1 (0.9)	2.1 ± 1.0 **NV**	[1.8, 2.4]	0.54 **MA**	2 (3.2)	1 (1.6)	1 (1.6)
Area they live in	1.7 ± 0.7 **NV**	[1.5, 1.8]	0.73 **SA**	–	–	2 (1.7)	1.9 ± 0.9 **NV**	[1.7, 2.2]	0.62 **MA**	1 (1.6)	–	1 (1.6)
Month of birth	1.2 ± 0.7 **NIA**	[1.1, 1.3]	0.75 **SA**	–	1 (0.9)	–	1.2 ± 0.7 **NIA**	[1.1, 1.5]	0.73 **SA**	–	–	–

ETM = estimated trim mean; EI = extremely important; VI = very important; I = important; NVI = not very important; NIA = not important at all); (95% CI), and within group agreement (interpretation (SA = strong agreement; MA = moderate agreement; WA = weak agreement; LoA = lack of agreement).

A total of 14 out of 17 (82%) psychological attributes were deemed as very important (estimated trimmed mean > 4.0) by coaches. Of those attributes, work rate (female program: *M* = 5.0, 95% CI [4.9, 5.0]; male program: *M* = 4.9, 95% CI [4.7, 5.0]) was perceived to have the highest mean importance (Table 3). But, when ranking the most important psychological attribute to consider during recruitment, coachability had the highest proportion in both female (30.2%) and male (32.3%) programs. Inter-rater reliability ranged from moderate to strong across coaches within both programs (*r*_wg _≥ 0.64 and ≤ 0.88). No significant differences were found in perceptions of importance for psychological attributes between coaches from female and male programs.

When considering technical attributes, coaches in both programs perceived ball control (female program: M = 4.6, 95% CI [4.5, 4.8]; male program: M = 4.5, 95% CI [4.3, 4.7]), receiving the ball (female program: M = 4.5, 95% CI [4.4, 4.6]; male program: M = 4.4, 95% CI [4.1, 4.6]), and technique (female program: M = 4.5, 95% CI [4.3, 4.6]; male program: M = 4.5, 95% CI [4.3, 4.7]), as the most important attributes for players to possess (Table 3). However, of those attributes, frequency analysis demonstrated that only ball control (43.1% vs. 40.3%) and technique (22.4% vs. 41.9%) were regularly ranked as the most important attributes in both the women’s and men’s programs, respectively. All perceptions of the importance of technical attributes demonstrated moderate to strong agreement (*r*_wg _≥ 0.51 and ≤ 0.90) in both the female and male programs. Only a moderate and significant difference in the perceived importance of finishing was found between coaches in both programs, with coaches in the female program perceiving finishing to have a higher mean importance (t = 2.83, p < 0.01, Mdiff = 0.3, 95% CI [0.1, 0.6], ξ = 0.32).

Decision-making had the highest mean importance of all attributes in both the female (M = 4.8, 95% CI [4.6, 4.9]) and male (M = 4.6, 95% CI [4.4, 4.8]) programs. This was supported by 55.2% and 54.8% of coaches working in the female and male game ranking decision-making as the most important attribute they considered during observation (Table 3). Further, all game intelligence attributes received a mean perceived importance rating of > 4.0 for coaches within female programs, with only creativity, visual search, and cue utilisation representing a mean perceived importance score of < 4.0 for coaches in male programs. Moderate to strong inter-rater agreement was shown between coaches in female (*r*_wg _≥ 0.67 and ≤ 0.85) and male (*r*_wg _≥ 0.62 and ≤ 0.78) programs. No significant differences were found on perceptions of the importance of game intelligence attributes between coaches from the female and male programs.

Coaches’ responses confirmed that teamwork, communication, being responsible, having adaptability, building relationships, accepting criticism, managing their lifestyle, and being prepared were all perceived to be very important social attributes (*M*≥ 4.0 and ≤ 4.8) when scouting players for both female and male programs (Table 3). However, when asked to rank the most important variables, a higher proportion of coach’s ranked adaptability (19.0%) as the most important attribute to consider when identifying female players, with coach’s identifying male players ranking teamwork as the most important attribute (19.4%). Inter-rater agreement amongst coaches within both programs showed moderate to strong agreement (*r*_wg _≥ 0.67 and ≤ 0.81). Moderate significant differences were found for communication (t = 2.87, p < 0.01, Mdiff = 0.4, 95% CI [0.1, 0.6], ξ = 0.32) and builds relationships (t = 3.10, p < 0.01, Mdiff = 0.5, 95% CI [0.2, 0.9], ξ = 0.48), with coaches in female programs perceiving these social attributes as more important to look for when recruiting than those in male programs.

Attributes outside the core performance domains (categorised as “other attributes”) received lower mean ratings of importance in both the female (*M*≥ 1.2 and ≤ 3.6) and male (*M*≥ 1.2 and ≤ 3.7) programs (Table 3) in comparison to attributes from physical, psychological, technical, game intelligence, and social domains. Innate talent was perceived as the most important variable (female program: M = 3.6, 95% CI [3.4, 3.7]; male program: M = 3.4, 95% CI [3.2, 3.6]) with coaches in female (31.0%) and male (27.4%) programs ranking this variable as the most important attribute. Agreement amongst other attributes was also more variable in comparison to attributes from core domains, with inter-rater agreement ranging from weak to strong (*r*_wg _≥ 0.39 and ≤ 0.75). A small but significant difference was found for the perceived importance of education level (t = −2.15, p < 0.01, Mdiff = −0.3, 95% CI [−0.6, 0.00], ξ = 0.23), with coaches in the male program perceiving this attribute as more important than those in the female program.

## Discussion

The current study describes evaluative priorities of college soccer coaches in the U.S. during the identification of talented youth male and female soccer players. Using a large sample of coaches, the findings make a significant contribution to the current understanding of the TI processes of college coaches, providing data on their perceived importance of various soccer-specific attributes from a soccer nation that is largely unexplored [66]. Data revealed that college-specific experience was rated as the most important attribute for TI coaches to possess across both programs (Table 2), and while male programs had a higher overall rating than females, coaching and scouting experience were rated as ‘very important’. This finding is not unexpected, as coaches in our study had a mean experience of 14.5 ± 10.4 years, with a large proportion being at the college level (13.2 ± 10.2 years) and likely influenced their perceptions. Previously, it has been shown that more-experienced soccer coaches employ different practice activities and behaviours [67], and visual search strategies during observation of soccer performance than their less-experienced counterparts (e.g., [68,69]). Yet this experience did not have an influence on how closely coaches subjective ratings matched comparative objective data [70]. It is currently unclear as to how and why college coaches perceive ‘experience’ as a key attribute in the TI process [41]. However, in a qualitative study examining combat sports (e.g., boxing) coaches reported that through experience their confidence and reliability in what to look for and what they could work with increased [71]. Future research could adopt a similar approach to gain a greater understanding of the cognitive strategies and rationales behind college soccer coaches’ responses (e.g., [72]).

Consistent with previous studies, soccer coaches perceived technical skills as highly important [25,30,42], with three skills (i.e., ball control, receiving the ball, and technique) being perceived as ‘extremely important’, and the remaining thirteen technical skills perceived as ‘very important’. Ball control (i.e., first touch) was the highest rated skill, which is not surprising, as coaches in Australia reported that scouts considered this to be a foundation for all other technical skills [30]. For instance, if a player has poor ball control, this can consequently affect their proceeding on-the-ball technical skills such as passing or shooting. Researchers have often assessed ball control via a ball juggling task (i.e., juggling a ball alternately with the left and right foot through as many subsections of a figure of eight-course without the ball touching the floor; [73]) that has shown to differentiate skill levels [74,75]. However, the transfer between this task and ball control in match-play is unknown [30,43]. Coaches also reported perceiving passing, running with the ball, and shooting as highly important, which is likely due to the requirement to be able to execute these actions to maintain possession and score a goal [25], but could also be due to the number of opportunities coaches have to observe and assess players technical skills within a game. For instance, there are 6–7 touches-per-possession in youth female soccer [76], and 602 releases-per-game in men’s college soccer [77]. This strong emphasis by coaches on technical attributes shows an alignment with scientific evidence, yet while researchers have shown that tests such as dribbling through cones, ball juggling and passing to a target can have predictive validity [8,10,78], they are often performed in isolation without the changing constraints of time. To provide objective information to support the subjective assessments of coaches [79], coaches have indicated that future research must utilise methodologies that are ecologically valid, where these skills are tested in-situ [4,30,80]. Furthermore, coaches perceived technique as extremely important, which allows the player to adapt and meet the demands of the dynamic and constantly changing situation, with constraints on time and space to perform the action [81]. It is likely that technique was rated very highly as it encompasses various skills, thus coaches predict talent holistically rather than specifically [43]. Only three technical skills were seen as important (e.g., blocking, intercepting, crossing), with previous studies reporting ‘crossing’ for midfield players was deemed as highly important (e.g., [25]), yet our survey lacked position-specific insights and is a limitation of the study.

Findings from this study underscore the perceived importance of game intelligence in college coaches’ TI, with decision-making emerging as the most valued attribute across both male and female programs. Over half of the coaches and scouts in each group ranked decision-making as the single most important attribute during recruitment. This aligns with prior research across TI ecosystems in Europe and Australia, where decision-making and broader game intelligence skills have been consistently prioritised over other attributes such as physical [25,30,43]. The consistently high rating across the category suggests that college coaches value these skills as fundamental to successful performance in soccer and is supported by the literature recognising decision-making as a key differentiator between elite and non-elite players in both lab-based and field-based settings [82,83]. Skilled soccer players consistently produce faster and more accurate anticipation and decision-making responses that are underpinned by task-specific visual search strategies [15,16,19,84]. Video-based decision-making tasks have been shown to reliably distinguish players at national, state, and sub-elite levels, reinforcing decision-making as an imperative predictor of future adult performance levels [85]. Despite the ability of game intelligence to differentiate future adult performance levels, there are currently few ecologically valid assessments [82]. While there have been recent attempts, such as using virtual reality [86] and/or 360-degree soccer environments [87], there remains a paucity of valid and reliable tools to accurately capture the constraints of game-representative environments, creating a gap between what stakeholders value and what they can robustly assess [43]. A potential avenue comes from a body of research examining tactical knowledge involving 3 vs. 3 scenarios and classifying behaviours based on offensive and defensive principles. Reviews have indicated high inter-rater reliability and a focus on real-game scenarios [88], however given coaches typically observe/identify players in live (i.e., 11 vs. 11) games, making this potentially logistically difficult. Moreover, while an absence of sex-based differences in perceptions may reflect a shared understanding of the cognitive demands of soccer, contextual factors such as playing-positions, formats, and player development histories may warrant further exploration. College soccer coaches rated a variety of physical attributes as important during the TI process. Notably, coaches identified pace and stamina as the most important physical attributes when assessing players. The multidimensional nature of soccer match play requires players to cover substantial distances (9.0–14.0 km) during a 90-minute game, interspersed with regular instances of explosive anaerobic activity, such as high intensity runs, sprints, and changes of direction [89,90]. Consequently, soccer players are typically expected to demonstrate competence in a variety of physical attributes to meet these demands. Early research identified measures of aerobic running performance as discriminatory between players of distinct performance standards [1,91]. However, due to the evolution of physical demands in soccer [92,93], greater emphasis has been placed on anaerobic skills when attempting to differentiate between players [43,94]. Research also suggests that high-speed running actions, such as linear and change of direction sprints, are commonly observed prior to goal scoring opportunities in soccer [95]. This evolution and increased importance of anaerobic qualities may also explain why coaches also considered pace to be the most important physical attribute to consider in both male and female programs. Interestingly, coaches from female programs perceived pace, agility, and accelerations significantly more important than coaches within male programs. Observational data from female soccer reports significantly lower total distances, but greater relative intensity (> 15 km/h) covered in comparison to male players [96,97]. These higher intensity bouts also typically represented a greater amount of match play, suggesting a higher playing tempo in female soccer. Although multidirectional speed qualities remain desirable in soccer holistically, our data suggest that coaches may particularly favour these attributes within female players. Finally, all attributes, except for jumping demonstrated strong inter-rater agreement for coaches working within male programs. The subjective nature of evaluation via utilisation of the ‘coach’s eye’ has previously been scrutinised due to its potential for disagreement between individuals [43,98]. While the observed agreement in what physical attributes should be assessed within the present study is positive, how scouts and coaches interpret and evaluate these physical qualities may vary [24]. Therefore, while coaches may agree on desirable attributes for soccer players, the complexity of subjective evaluations should be recognised, particularly between individuals.

Results highlighted a strong consensus among college soccer coaches regarding the critical importance of psychological attributes in TI, with no statistically significant sex-based differences in perceptions between coaches in male and female programs. This is particularly notable given previous concerns in the literature regarding the underrepresentation and under-assessment of psychological characteristics in formal TI frameworks [25,29]. Participants rated 82% of the psychological attributes as ‘very important’ (*M* > 4.0), underscoring a shift in TI practices toward recognising the central role of psychological resilience and adaptability in elite development. The highest mean importance was attributed to work rate (female programs: *M* = 5.0; male programs: *M* = 4.9) and aligned with earlier research that has consistently emphasised motivation and effort as predictors of long-term success [25,27]. Still, when asked to rank the single most important psychological trait, coaches prioritised coachability, emphasising a preference for players who are either responsive to instruction, capable of adapting to the demands of collegiate sport, or have inclination for learning. The consistency of inter-rater agreement (*r*_wg_ range = 0.64–0.88) further supported the robustness of these perceptions across coaching cohorts. Suggesting that, despite historic difficulties in operationalising and measuring psychological traits [25], coaches may have converged on a shared conceptual understanding of their value in player assessment.

These results also reflect broader concerns regarding contextual dependencies of psychological trait development. Indeed, attributes such as motivation and emotional regulation can be shaped by socio-economic conditions, access to coaching, and even birthplace effects [37,39,99]. Consequently, while coaches rightfully acknowledge the value of psychological factors, it is crucial that their assessments are nuanced and contextualised. For instance, traits like resilience may manifest differently depending on an athlete’s lived experiences or access to support systems [27]. This underscores the importance of avoiding rigid, one-dimensional interpretations of psychological readiness. Moreover, the prioritising of psychological factors in this study may reflect the unique context of college soccer in the U.S. Unlike academy-based systems common in Europe, college recruitment often occurs at later developmental stages, when players are expected to be performance ready [47,53]. In this environment, psychological maturity may be perceived not merely as a developmental asset, but as a precondition for success. Coachability may be favoured as it signifies a player’s capacity to integrate quickly into a structured environment with high expectancies and limited adaptation time. These findings reinforced earlier calls for more systematic, evidence-informed approaches to the evaluation of psychological traits [25]. While coach perceptions are valuable, especially when consistent across large samples, integrating structured assessments (i.e., behavioural interviews, validated psychometric tools) may further enhance the reliability of these evaluations. This is particularly pertinent as psychological attributes interact with technical, tactical, and sociological skills to form the complex profiles that underpin soccer potential.

Coaches’ ratings indicated that all social attributes were perceived as either ‘very important’ or ‘extremely important’ with moderate to strong agreement across both male and female programs. These findings may reflect the growing recognition of the need for holistic athlete development within talent development environments [100–102], where psychosocial skills are recognised as essential for long-term success [27,103], regardless of sex. Supporting this, research in youth soccer suggests that player’s perceptions of positive social relationships, peer acceptance, and friendship quality are strongly associated with soccer continuation, further highlighting the importance of social predictors for soccer participation and performance [104]. Notwithstanding the convergence in attribute ratings, significant differences were found for ‘communication’ and ‘building relationships’, with these attributes being favoured more in female soccer players. This mirrors previous findings that female athletes, particularly during mid-adolescence, demonstrate a stronger need for social cohesion and closer peer relationships [105]. Coaches may tacitly discern this and be intuitively responding to this need as reflected through their rated perceptions. The greater emphasis on ‘communication’ in the female program may also stem from differences in the coach-athlete dyad, with research suggesting potential differences between female and male perceptions of the coach-athlete relationship [101,106,107]. High quality coach-athlete relationships are known to be positively correlated with athletes’ developmental experiences [108], and female athletes often place greater value on frequent and positive communication and expect coaches to be aware of sex dynamics [107]. Similarly, LaVoi’s [109] findings demonstrate that female NCAA athletes prioritise communication in the coach-athlete relationship more than their male counterparts. These findings may be partially explained with evidence that female athletes prefer coaches of the same sex due to their communication styles and approachability [106], and that sex dynamics in coach-athlete relationships can create power imbalances, particularly when male coaches work with female athletes [110]. Sex differences also emerged in the ranking of the most important social attributes. Specifically, adaptability was most frequently ranked first in female recruitment, consistent with findings that self-regulatory and volitional behaviours are key in female player development [101]. In contrast, male coaches ranked teamwork highest, reflecting that belonging to a sport team establishes strong ties within a social group, with evidence suggesting strong social cohesion is linked to sporting success [111].

Within this study, other attributes revealed their relatively lower prioritisation in the TI process compared to other domains. This aligns with previous research that has indicated that certain non-performance-based characteristics (e.g., educational level or innate talent) may play a supplementary role in player assessment, they are often secondary to more observable and sport-specific qualities [25,30]. Among these attributes, innate talent emerged as the most highly valued by coaches in both female (*M* = 3.6) and male (*M* = 3.4) programs, with over 30% and 27%, respectively, ranking it as the most important attribute within this category. This suggests that while coaches predominantly value observable skills, there remained an underlying belief in a natural ability or predisposition to excel, particularly at the collegiate level where athletes must transition rapidly into performance-ready roles. The recognition of innate talent may reflect lingering essentialist views about athletic potential, even as research increasingly emphasises developmental opportunity and environmental shaping over fixed periods [12,13]. Interestingly, educational level was the only attribute with a statistically significant program difference (*p* < 0.01), with coaches in male programs assigning slightly more importance to academic credentials, which could be indicative of varying recruitment cultures or program demands. For example, male coaches may view educational attainment as a proxy for discipline or time management, especially in the context of the U.S. collegiate system, which places unique dual demands on student-athletes [50]. Alternatively, this difference may reflect varying institutional expectations or scholarship structures that influence coach priorities differently across male and female programs. Despite these nuances, the overall lower ratings (M^range^ = 1.2–3.7) and variable inter-rater agreement (*r*_wg_ = 0.39–0.75) across all other attributes suggested these factors are neither central nor consistently interpreted within the TI process. This aligns with the critique that such attributes, particularly educational level and other demographic indicators, might be inconsistently weighed and vulnerable to subjective or biased interpretation [37,99]. The relatively weak agreement across coaches further implies a lack of shared conceptual clarity about how or whether these traits should meaningfully inform selection decisions.

This raised two important considerations. Firstly, the persistent, albeit marginal, influence of innate talent beliefs warrants attention. Assumptions about natural ability can obscure the critical role of structured training environments and developmental opportunities [2]. Specifically, within diverse populations, reliance on perceived talent, absent reliable operational definitions, risks reinforcing existing biases or overlooking late-developing players [4]. Second, the inclusion of educational level as a relevant albeit minor consideration, underscored the distinctive nature of collegiate recruitment. Distinct from European academies, U.S. college programs operate within an academic framework, and coaches may need to balance athletic promise with academic eligibility and institutional fit [52]. While other attributes were not prioritised to the same degree as technical, psychological, or game-related attributes, their presence in coaches’ evaluative frameworks reflects the complex, context-dependent nature of TI in college soccer. These attributes may offer additional insight when interpreted cautiously and contextually, but their low consensus and variable perceived value underscore the need for clearer guidelines if they are to inform selection decisions meaningfully.

### Limitations

While our study provides a contribution of our understanding on how college soccer coaches in the U.S. perceive and prioritise attributes related to TI, limitations should be acknowledged that may influence the interpretation and generalisability of the findings. Firstly, the study employed a non-randomised, convenience sampling approach supplemented by snowball sampling. As a result, the sample may not fully represent the broader population of college coaches in the U.S., particularly those working in smaller programs and/or outside of the NCAA. Second, although the sample included coaches from both male and female programs, the sex distribution was imbalanced, with no male-identifying coaches represented in women’s teams and no female-identifying coaches in men’s teams. This separation may reflect structural realities of coaching demographics, but it limits the ability to disentangle whether observed differences in attribute prioritisation are due to the sex of the coach, program, or a combination of both. Future studies employing a more balanced and diverse sample of coaches across roles and program types would provide greater nuance to sex-related analysis. Third, the study relied exclusively on coaches’ perceptions as a method for TI. Research has demonstrated that such methods can be imperfect and often subject to a number of (sub)conscious cognitive biases (e.g., use of heuristics, conformation bias, endowment effect; [112,113]. Coaches may have responded in ways that reflect aspirational rather than actual practice. While Likert-scale ratings and frequency analysis offer useful descriptive insights, they do not capture the contextual nuances or underlying rationale behind specific evaluative decisions. Further, due to the lack of standardised definitions provided for coaches, conceptual ambiguity may have led to biased responses and/or increased variability in coach responses. Therefore, despite moderate to strong inter-rater agreement and relatively low within-group variability (SD), effect sizes and inter-rater variability should be interpreted with caution as they may reflect differences in conceptual understanding that could inflate or obscure statistical interpretation. As such, the survey data provide only a partial window into the complex, often intuitive judgments that underpin scouting and recruitment. For example, whilst the current study asked coaches for their perceptions of individual attributes, many of these attributes do not occur and are not observed in isolation, with research suggesting coaches typically combine information from multiple attributes to make holistic judgements [43,71]. Additionally, the survey, although adapted from a previously utilised instrument [25], did not account for positional specificity. Skills such as finishing, pace, or decision-making may vary in importance depending on the playing-position observed (e.g., goalkeeper vs. midfielders), yet the instrument treated all evaluations as position-neutral [25,114]. This may have diluted the precision of the data and limited the ability to detect role-specific patterns in coach perceptions. Finally, the study’s cross-sectional design captures perceptions at a single point in time and cannot account for how coach priorities might evolve across recruitment cycles, changes in institutional policies, or broader shifts in the college soccer landscape. Longitudinal research would help to better understand the stability or malleability of TI perceptions over time and in response to contextual pressures such as rule changes, resource constraints, or performance outcomes. Taken together, these limitations highlight the importance of interpreting the findings as indicative rather than definitive. They also underscore the need for continued research that could help validate the importance of the attributes from the current study, for example, the use of predictive models and the incorporation of mixed-methods and longitudinal and multidimensional approaches, along with more inclusive sampling frameworks [2–4]. Such research would help build a more comprehensive understanding of how talent is identified and evaluated in college soccer and possible interactions between predictive variables.

## Conclusion

This study provides insights into the perceptions of NCAA DI and DII college soccer coaches regarding the attributes and methods they consider most important when identifying talent across male and female programs in the U.S. The results demonstrated a high degree of consensus among coaches, particularly value placed on technical skills, psychological resilience, decision-making and broader game intelligence skills, and live game observation. Notably, college-specific soccer knowledge and coachability emerged as central priorities, suggesting a performance-ready TI process aligned with the structural demands of collegiate sport. Differences between coaches in male and female programs were generally limited in magnitude, but where they did occur, such as in perceptions of physical and social attributes, they highlighted subtle variations that may reflect distinct contextual or developmental priorities across sex environments. Coaches in female programs, for example, placed more emphasis on communication and relationship-building, echoing broader research on the psychosocial needs of female athletes. Equally, the continued presence of innate talent as a meaningful, yet secondary, consideration pointed to the persistence of essentialist beliefs that warrant further interrogation in practice and policy. Importantly, the emphasis placed on decision-making by coaches underscores its relevance as a key differentiator, reinforcing calls for talent systems to better integrate the assessment and development of game intelligence skills within recruitment processes. Whilst no ‘gold-standard’ approach exists for TI, the findings of this study reinforce the multidimensional nature of TI in soccer, providing insight into current norms for TI in US college soccer. The study underscored the importance of contextualising these perceptions within the unique landscape of U.S. collegiate sport, which differs markedly from other talent systems found elsewhere. As such, understanding the rationale behind coach decision-making remains vital for developing more equitable, evidence-informed, and developmentally supportive TI processes. Future research should build on these insights by employing mixed method approaches, accounting for position-specific demands, and exploring longitudinally coach priorities. Doing so will help bridge the gap between what coach’s value, what can be measured, and what ultimately supports player development and success within and beyond the collegiate setting.

## Supporting information

S1 Data (XLSX)
